# Medical Society of the State of New York

**Published:** 1912-04

**Authors:** 


### Medical Society of the State of New York.

THE 106th Annual Meeting of the Medical Society of the State of New York will be held at Albany, Tuesday, Wednesday and Thursday, April 16-18, 1912. Tuesday morning there will be the addresses of welcome, etc., followed by a half dollar luncheon at German Hall. This year, the scientific sessions will be by sections, Medicine, Surgery, Mental and Nervous Diseases, etc., Public Health and Eye-Ear-Nose-Throat, each of which will hold one session Tuesday and two each on Wednesday and Thursday, except that the Medical and Surgical Sections will meet together Thursday morning for symposiums on Infantile Paralysis and Hyperthyroidea and that The Medical and Specialty Sections will meet together Wednesday afternoon for a symposium on Vertigo.

Tuesday evening, there will be a public meeting in the Assembly Chamber, with addresses on Prophylaxis as follows: Blindness, Dr. George E. de Schweinitz of Philadelphia; Deafness, G. Hudson Makuen of Philadelphia; Insanity, Albert Warren Ferris of Watkins; Tuberculosis, Homer Folks representing the State Charities Aid Association, of New York.

Wednesday at 2 o'clock, John M. T. Finney of Baltimore, will deliver the surgical oration on the Duty of the Family Physician in the Management of Surgical Cases. At 8 o'clock, Walter B. Cannon will deliver an oration on the Benefits of Vivisection. At 9 o'clock, there will be the President's reception, dance and supper at the Hotel Ten Eyck ($2).

We regret that space is lacking for the entire program whose greatest value lies, as usually, in the routine papers presented to the sections.

---

THE Buffalo General Hospital Internes held their annual banquet March 10, at the Lafayette Hotel. Dr. J. W. Putnam delivered an address on Expert Testimony. Dr. DeLancey Rochester was toastmaster, and among those who responded were Dr. H. U. Williams, Dr. Clayton M. Greene, and Dr. E. L. Shurley of Detroit.

---

The Buffalo Medical and Surgical League was organized on Thursday night, March 14, at the Lafayette Hotel. Officers were chosen as follows: President, Dr. A. W. Hengerer; vice-president, Dr. M. Allen Richter; secretary, Dr. Julius Richter; treasurer, Dr. Edmund P. Reiman; board of governors, Dr. George A. Stesel, chairman; Dr. Francis O'Gorman, Dr. Robert S. Taylor, Dr. Wilhelm Brauns, Dr. George Seitz. The promulgation of the code of ethics of the American Medical Association is one of the objects for which the league is formed.

